# Timeline of changes in adaptive physiological responses, at the level of energy expenditure, with progressive weight loss

**DOI:** 10.1017/S0007114518000922

**Published:** 2018-05-07

**Authors:** Siren Nymo, Silvia R. Coutinho, Linn-Christin H. Torgersen, Ola J. Bomo, Ingrid Haugvaldstad, Helen Truby, Bård Kulseng, Catia Martins

**Affiliations:** 1 Obesity Research Group, Department of Clinical and Molecular Medicine, Faculty of Medicine, Norwegian University of Science and Technology, Prinsesse Kristinas veg 5, 7030 Trondheim, Norway; 2 Centre for Obesity and Innovation (ObeCe), Clinic of Surgery, St. Olav University Hospital, Prinsesse Kristinas veg 5, 7030 Trondheim, Norway; 3 Department of Nutrition, Dietetics & Food, Monash University, Melbourne, 264 Ferntree Gully Road, Notting Hill, VIC 3168, Australia

**Keywords:** Adaptive thermogenesis, RMR, Exercise-induced energy expenditure

## Abstract

Diet-induced weight loss (WL) is associated with reduced resting and non-resting energy expenditure (EE), driven not only by changes in body composition but also potentially by adaptive thermogenesis (AT). When exactly this happens, during progressive WL, remains unknown. The aim of this study was to determine the timeline of changes in RMR and exercise-induced EE (EIEE), stemming from changes in body composition *v*. the presence of AT, during WL with a very-low-energy diet (VLED). In all, thirty-one adults (eighteen men) with obesity (BMI: 37 (sem 4·5) kg/m^2^; age: 43 (sem 10) years) underwent 8 weeks of a VLED, followed by 4 weeks of weight maintenance. Body weight and composition, RMR, net EIEE (10, 25 and 50 W) and AT (for RMR (AT_RMR_) and EIEE (AT_EIEE_)) were measured at baseline, day 3 (2 (sem 1) % WL), after 5 and 10 % WL and at weeks 9 (16 (sem 2) %) and 13 (16 (sem 1) %). RMR and fat mass were significantly reduced for the first time at 5 % WL (12 (sem 8) d) (*P*<0·01 and *P*<0·001, respectively) and EIEE at 10 % WL (32 (sem 8) d), for all levels of power (*P*<0·05), and sustained up to week 13. AT_RMR_ was transiently present at 10 % WL (−460 (sem 690) kJ/d, *P*<0·01). A fall in RMR should be anticipated at ≥5 % WL and a reduction in EIEE at ≥10 % WL. Transient AT_RMR_ can be expected at 10 % WL. These physiological adaptations may make progressive WL difficult and will probably contribute to relapse.

Obesity, owing to its high prevalence, associated co-morbidities and large socio-economic costs^(^ ^1^ ^)^, is probably one of the largest public health problems of the 21st century. Even though a modest weight loss (WL) of 5–10 % is sufficient to induce health benefits^(^ ^2^ ^)^ and can be achieved in the short term (3–6 months), 80 % will experience relapse, with weight regain apparent after 6–12 months^(^ ^3^ ^,^ ^4^ ^)^, making WL maintenance a substantial unresolved issue.

The reduced obese state is associated with increased appetite^(^ ^5^ ^–^ ^7^ ^)^ that fuels the desire to consume more energy, despite an overall reduction in total energy expenditure (EE), attributable to a reduction in both resting and non-resting EE, mainly driven by the loss of metabolic active tissue^(^ ^8^ ^,^ ^9^ ^)^. The reduction in non-resting EE seen with WL seems to be accounted for mainly by a reduction in exercise-induced EE (EIEE)^(^ ^8^ ^,^ ^9^ ^)^, probably owing to increased efficiency^(^ ^10^ ^)^, given that physical activity (PA) levels have been shown to increase or not to change with sustained WL^(^ ^11^ ^,^ ^12^ ^)^. Increased skeletal muscle work efficiency means that less energy is used to perform the same volume of exercise^(^ ^10^ ^)^. Moreover, some^(^ ^8^ ^,^ ^10^ ^,^ ^13^ ^,^ ^14^ ^)^, but not all, studies^(^ ^15^ ^,^ ^16^ ^)^ report a reduction in total EE and its components (resting and non-resting EE) in excess of what would be predicted, given the measured alterations in fat mass (FM) and fat-free mass (FFM), a mechanism known as adaptive thermogenesis (AT). Therefore, AT can account for a small proportion on the reduction in EE seen with WL. The extent to which these different, but inter-related, physiological mechanisms are important remains controversial. However, combined, these mechanisms may act to reduce WL rate and increase the risk of weight re-gain^(^ ^7^ ^)^.

AT, which is induced by conditions of negative energy balance, has been shown to be under the influence of several hormones and the sympathetic nervous system. Thyroid hormones, insulin and leptin, as well as sympathetic activity, are likely to be involved in the greater than predicted reduction in both resting and non-resting EE observed with WL^(^ ^17^ ^)^. At a cellular level, mitochondrial adenosine triphosphate synthesis efficiency and uncoupling proteins are likely to be involved^(^ ^17^ ^,^ ^18^ ^)^.

To our knowledge, no studies have determined the timeline over which EE, both at rest and during exercise, changes with progressive WL in the obese population. A minimal, but significant, WL (1–2 kg) has been shown to reduce RMR, even below predicted values (AT) in some studies^(^ ^13^ ^)^, whereas others report no change^(^ ^19^ ^)^. A reduction in EIEE has been reported after 5 % and 10 % WL (10–13 kg)^(^ ^10^ ^,^ ^20^ ^,^ ^21^ ^)^, in some cases below predicted values (AT)^(^ ^21^ ^)^, whereas others have reported no change even after a 19 % WL^(^ ^22^ ^)^. The results are clearly controversial and more research is needed. Moreover, the greater FFM content of WL during energy restriction in men, compared with women^(^ ^23^ ^)^, may suggest that the changes in EE variables with progressive WL are modulated by sex. Therefore, the primary aim of this study was to determine the timeline over which changes in EE variables (RMR, EIEE and AT) occur during progressive WL with a very-low-energy diet (VLED). A secondary aim was to assess whether this timeline was modulated by sex.

## Methods

### Participants

Healthy adults (18–65 years of age) with obesity (30≤BMI <45 kg/m^2^) were recruited from the local community by means of newspaper and internet advertising. This study nests within a large WL intervention (*n* 100), where individuals with obesity undertook 8 weeks of a VLED and were followed up to 1 year.

Inclusion criteria were as follows: weight stability (<2 kg change over the last 3 months), not dieting to lose weight and an inactive lifestyle (defined as <150 min of PA of at least moderate intensity^(^ ^24^ ^)^, which was corroborated via data from the SenseWare activity data collected at baseline; see more details below). Owing to the known effect of phase of menstrual cycle on RMR^(^ ^25^ ^)^, women had to be post-menopausal or taking hormonal contraceptives. Exclusion criteria were pregnancy, breast-feeding, clinical significant illness, including diabetes, previous WL surgery and medication known to affect appetite/metabolism or induce WL.

### Ethical statement

The study was approved by the regional ethical committee (reference 2012/1901), registered in ClinicalTrial.gov (NCT01834859) and conducted according to the Declaration of Helsinki, with all participants providing informed written consent.

### Study design

**Fig. 1:**
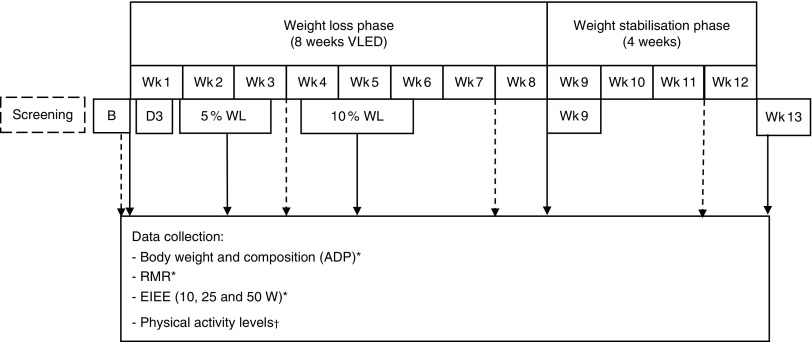
Study diagram. For data collection points, see arrows. Wk, weeks; VLED, very-low-energy diet; ADP, air-displacement plethysmography; EIEE, exercise-induced energy expenditure. * 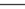; † 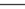.

This was a clinical intervention study with repeated measurements. All participants underwent a supervised VLED for 8 weeks, followed by 4 weeks of weight stabilisation, and were asked not to change their PA levels throughout the study (see Fig. 1).

### Weight-loss phase

Participants followed for 8 weeks a VLED (Allévo; Karo Pharma AS) with 2·3/2·8 MJ/d, for women and men, respectively (carbohydrates 42 %, protein 36 %, fat 18 % and fibre 4 %), as well as no-energy fluids and low-starch vegetables (max 100 g/d).

### Weight stabilisation phase

At week 9 (Wk9), participants were gradually introduced to normal food, and an individual diet plan was prescribed by a trained dietitian based on estimated energy requirements (measured RMR×PAL (from individual SenseWear data at week 8)), with 15–20 % energy provided by protein, 20–30 by E% fat and 50–60 E% by carbohydrates, tailored to achieve weight stabilisation^(^ ^26^ ^)^.

### Objective measures of compliance

#### Diet

Participants received a weekly follow-up face-to-face consultation with a dietitian, which included measuring body weight, review of daily food records and monitoring of side effects. Urine acetoacetic acid concentration was measured weekly, using Ketostix reagent strips^®^. Negative ketones (<0·5 mmol/l) more than once were the reasons for exclusion from the analysis.

#### Physical activity

Armbands (BodyMedia^®^; SenseWare) were used for 7 d at baseline, and at weeks 4, 8 and 12. The data were considered valid if the participants wore the device for ≥4 d, including at least 1 weekend day, >95 % of the time^(^ ^27^ ^)^. The following variables were analysed: average metabolic equivalent of task (MET), time spent on sedentary, light, moderate and vigorous activities, total PA duration and steps/d.

### Data collection

The following measurements were conducted at baseline, day 3, when each individual participant reached 5 and 10 % WL, and at week 9 (the day immediately after the end of the VLED) and week 13 (Wk13).

#### Body weight and body composition

Air-displacement plethysmography (ADP) (BodPod; COSMED) was used while participants were in fasting state and in accordance with standard operating procedures.

#### RMR

RMR was measured in fasting state by indirect calorimetry (*V*_max_ Encore 29N; CareFusion) using a canopy system and following standard procedures^(^ ^28^ ^)^. Participants were asked to fast for 12 h, not to drink caffeine for at least 6 h, be nicotine abstinent over the last 2 h and not to perform moderate-intensity PA for 2 h before test. Although calibration of the equipment was performed, the participants rested for 10 min on a chair. Thereafter, a ventilation hood was placed over the person’s head, and VO_2_ and CO_2_ production (VCO_2_) were measured for 15–20 min (or longer if required) until ‘steady state’ was reached. The first 5 min were excluded, and 10 min of stable data (CV for VO_2_ and VCO_2_<10 %) were used^(^ ^28^ ^)^.

#### Exercise-induced energy expenditure

EIEE was measured by graded cycle ergometry (Eromedic 839E, GIH; Monark), 3 h after a standardised meal (2·5 MJ: 17 % protein, 35 % fat and 48 % carbohydrates). Participants pedalled at 60 rpm against graded resistance to generate 10, 25 and 50 W of power in sequential 4-min intervals. Gas exchange was measured continuously using a face mask by indirect calorimetry (*V*_max_ Encore 29 N), and the average of the last 2 min at each stage was used for analysis. Net EIEE was calculated by subtracting RMR (kJ/min) from the gross EIEE^(^ ^21^ ^)^.

### Adaptive thermogenesis

AT was present when measured EE (RMR or EIEE) was lower than predicted, given the body composition (FM and FFM) measured at each time point.

Regression analysis was performed to develop equations to predict both RMR (RMRp) and net EIEE (EIEEp) at each time point, using body composition (FM and FFM (kg)), sex, age and height as predictors. Equations to predict RMR and net EIEE were derived from a data set of ninety-nine participants (forty-four male, aged 43 (sem 10) years with a BMI of 36 (sem 4) kg/m^2^), which this study is a part of (the participants included in this study were part of the data set):
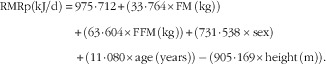

*R* ^2^=0·78, sem=591 kJ/d and *P*<0·001.
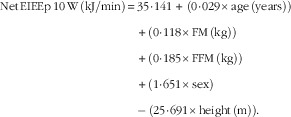

*R* ^2^=0·47; sem=2·10 kJ/min and *P*<0·001.
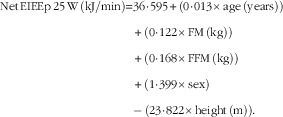

*R* ^2^=0·45; sem=2·19 kJ/min and *P*<0·001.
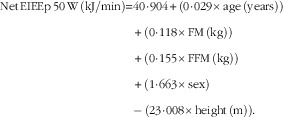

*R* ^2^=0·36; sem=2·41 kJ/min and *P*<0·001.

### Power calculation

Sample size estimation was based on expected changes (from baseline) in RMR (day 3: −209; 5% WL: −419, 10% WL: −544, Wk9: −670 and Wk13: −377 kJ/d)^(^ ^13^ ^,^ ^19^ ^,^ ^29^ ^,^ ^30^ ^)^ for a repeated-measures design. For an sd of 958 kJ/d^(^ ^30^ ^)^, at a power of 80 %, a significance level of 5 % and assuming a 30 % correlation between time points, thirty-two participants were needed.

### Statistical analysis

Statistical analysis was performed with SPSS version 22 (SPSS Inc.), and data were presented as means with their standard errors, except for baseline anthropometric data, time to achieve 5 and 10 % WL and WL (%) at day 3, Wk9 and Wk13, where means and standard deviations are presented. Statistical significance was set at *P*<0·05. Data were analysed using linear mixed-effects models, with restricted maximum-likelihood estimation, including fixed effects for time and sex, and their interaction. Bonferroni correction was used for post-hoc pairwise comparisons. RMR was also adjusted for FM and FFM (RMR_adj_) and analysis was performed by linear mixed-effects models (LMM). Participants with at least three time points were considered completers and kept in the analysis. The Benjamini–Hochberg method, which controls for the false discovery rate^(^ ^31^ ^)^, was used to adjust for the number of outcome variables.

The presence of AT was tested by paired *t* tests, comparing measured and predicted variables (RMR and EIEE), and a *P*<0·003 was considered significant after correcting for multiple comparisons. Correlation analysis was performed between WL and AT_RMR_ and AT_EIEE_.

The data sets used and/or analysed during the present study are available from the corresponding author on reasonable request.

## Results

### Participants

A total of thirty-three Caucasian participants started the study and thirty-one (eighteen males) were included in the analysis (one woman withdrew owing to personal reasons and one man owing to not tolerating the VLED). Completers had a BMI of 36·7 (sem 4·5) kg/m^2^ and were 43 (sem 10) years of age. Women had significantly lower body weight (102·7 (sem 16·3) *v*. 124·1 (sem 18·1) kg, *P*<0·01) and FFM (55·6 (sem 9·1) *v*. 74·2 (sem 11·6) kg *P*<0·001) compared with men, but there were no significant differences in BMI between sexes.

### Compliance

#### Diet

Compliance with the VLED was excellent, with no participant being excluded on the basis of not being ketotic.

#### Physical activity

No significant changes in any of the PA variables analysed were found^(^ ^32^ ^)^.

### Body weight and composition

**Fig. 2:**
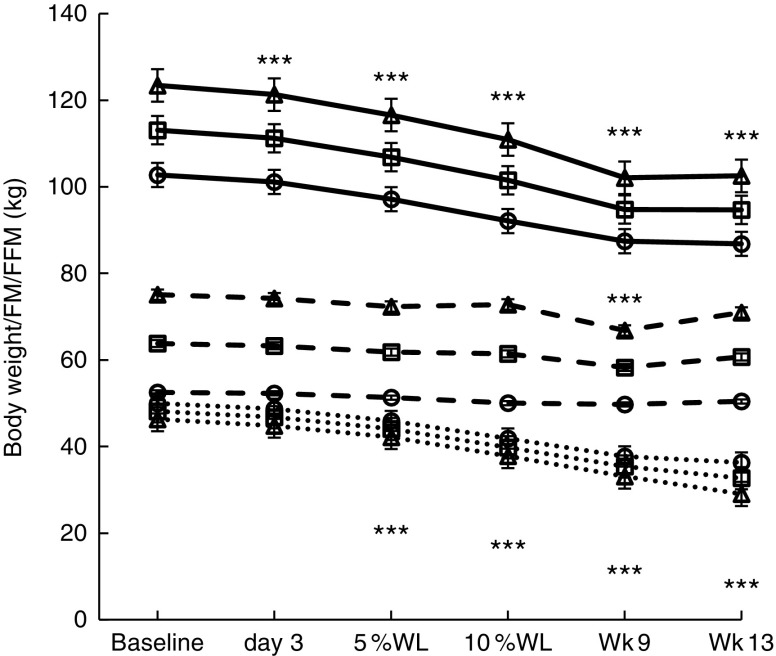
Body weight and composition over time in all participants, men and women, with progressive weight loss. Values are estimated marginal means with their standard errors. Wk9, week 9; Wk13, week 13; WL, weight loss; FM, fat mass; FFM, fat-free mass; □, all participants; ∆, males; ○, females; 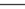, body weight; 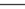, FFM; 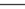, FM. Significant differences from baseline in all participants: ***P<0·001 for body weight, FFM and FM.

Changes in body weight/composition are reported in Fig. 2. Significant WL (kg) occurred by day 3 (*P*<0·001) in all participants and in males (1·9 (sem 0·9) and 2·1 (sem 1·1) kg, respectively), which continued until Wk9 in all participants (18·7 (sem 4·1) kg, *P*<0·001), and then stabilised (19·2 (sem 3·4) kg, *P*<0·001). On average, participants achieved 5 % WL in 12 (sem 6) d (11 (sem 5) and 15 (sem 7) d, for men and women, respectively, NS) and 10 % WL in 32 (sem 8) d (28 (sem 7) and 37 (sem 6) d, for men and women, *P*<0·01). Men lost significantly more weight than women overall (12·8 (sem 0·4) *v*. 10·0 (sem 0·4) kg, *P*<0·05).

FM (kg) was significantly reduced by 5 % WL in all participants, men (*P*<0·001 for both) and women (*P*<0·01), and continued to decrease with progressive WL, being lower than baseline at all time points from WL≥5 %. FM loss at Wk9 (16 % WL) was significant in all groups (12·8 (sem 0·8), 13·2 (sem 1·1) and 12·3 (sem 1·2) kg, *P*<0·001 for all). A significant loss of FFM was seen at Wk9 only (16 % WL) in all participants and in males (5·2 (sem 1·0) and 8·3 (sem 1·4) kg, *P*<0·001 for both) (no significant changes were seen in females at any time point). Women had a significantly lower overall FFM compared with men (51·0 (sem 1·9) *v*. 72·1 (sem 1·6) kg, *P*<0·001).

### RMR

RMR (kJ/d) was significantly reduced after 5 % WL in all participants (674 (SEM 121) kJ/d, *P*<0·001), men (770 (SEM 159) kJ/d, *P*<0·001) and women (574 (SEM 188) kJ/d, *P*<0·05), and further WL did not alter it significantly (see Table 1). RMR was lower than baseline at all time points, except for women at Wk13, where RMR was no longer different from baseline. No significant changes in absolute RMR were seen between Wk9 and Wk13, except in males where an increase was seen (*P*<0·01), even though values at Wk13 were still below baseline (*P*<0·05). RMR was significantly higher in men overall (7046 (SEM 197) *v.* 5347 (SEM 230) kJ/d, respectively). Adjusted RMR (kJ/d) was only significantly lower than baseline at 5 and 10 % WL in all participants (*P*<0·01, for both), and 10 % and Wk9 (16 % WL) in men (*P*<0·01 and *P*<0·05, respectively). A significant increase in adjusted RMR was seen between Wk9 and Wk13 (*P*<0·01) in men only.

**Table 1:** RMR over time in all participants, men and women (Mean values with their standard errors)

	Baseline		Day 3		5 %WL		10 %WL		Wk9		Wk13	
	Mean	sem	Mean	sem	Mean	sem	Mean	sem	Mean	sem	Mean	sem
RMR (MJ/d)												
All	6·8	0·2	6·6	0·2	6·1***	0·2	5·9***	0·2	5·8***	0·2	6·1***	0·2
Men	7·7	0·2	7·6	0·2	6·9***	0·2	6·6***	0·2	6·3***	0·2	7·1*	0·2
Women	5·8	0·3	5·6	0·3	5·2*	0·3	5·1**	0·3	5·2*	0·3	5·1	0·3
RMR_adj_ (MJ/d)												
All	6·5	0·1	6·4	0·1	6·0**	0·1	5·9**	0·1	6·0	0·1	6·4	0·2
Men	7·0	0·2	7·0	0·2	6·5	0·2	6·3**	0·2	6·3*	0·2	7·1	0·2
Women	5·9	0·2	5·8	0·2	5·5	0·2	5·5	0·2	5·8	0·2	5·7	0·3

WL, weight loss; Wk9, week 9; Wk13, week 13; RMR_adj_, RMR adjusted for fat-free mass and fat mass as covariates in LMM.

Significant differences from baseline: * *P*<0·05, ** *P*<0·01, *** *P*<0·001.

Adjusted RMR was significantly higher in men overall (6703 (sem 155) *v*. 5690 (sem 180) kJ/d, respectively, *P*<0·001).

### Net exercise-induced energy expenditure

Net EIEE at 10 W was significantly reduced, compared with baseline, after 10 % WL in all participants (*P*<0·01) and in males (*P*<0·05), and remained significantly lower than baseline at Wk9 (*P*<0·001 and *P*<0·01, respectively) and Wk13 (*P*<0·001 for both) (see Table 2). In women, a significant reduction in net EIEE at 10 W was seen at Wk13 (*P*<0·01). Net EIEE at 25 W was significantly reduced for the first time at 10 % WL in all participants (*P*<0·01), and in men at Wk9 (*P*<0·01) and continued to be lower afterwards (*P*<0·001, for both all and males). Net EIEE at 50 W was significantly reduced at 10 % WL in all participants and in males (*P*<0·05 for both), and remained lower than baseline at Wk9 (*P*<0·01 for both) and Wk13 (*P*<0·001 for both), but no differences between Wk9 and Wk13 were seen for any groups.

**Table 2:** Net exercise-induced energy expenditure (EIEE) over time in all participants, men and women† (Mean values with their standard errors)

	Baseline		Day 3		5 %WL		10 %WL		Wk9		Wk13	
	Mean	sem	Mean	sem	Mean	sem	Mean	sem	Mean	sem	Mean	sem
Net EIEE 10 W (kJ/min)												
All	11·93	0·50	11·30	0·50	10·93	0·50	10·47**	0·50	10·22***	0·50	9·21***	0·54
Men	12·48	0·63	11·68	0·63	11·47	0·63	10·97*	0·67	10·38**	0·67	9·67***	0·71
Women	11·35	0·75	10·89	0·75	10·34	0·75	10·01	0·75	10·01	0·75	8·71**	0·88
Net EIEE 25 W (kJ/min)												
All	14·82	0·54	14·03	0·54	13·82	0·54	13·36**	0·54	13·10**	0·54	12·31***	0·59
Men	15·41	0·67	14·44	0·64	14·49	0·67	13·86	0·67	13·40**	0·71	12·73***	0·75
Women	14·24	0·80	13·61	0·80	13·60	0·80	12·90	0·80	12·81	0·80	11·93	0·96
Net EIEE 50 W (kJ/min)												
All	20·22	0·54	19·68	0·54	19·85	0·54	18·71*	0·54	18·51**	0·54	17·50***	0·59
Men	20·72	0·67	20·10	0·71	20·47	0·67	18·92*	0·71	18·55**	0·71	17·79***	0·75
Women	19·72	0·84	19·22	0·84	19·05	0·80	18·15	0·80	18·51	0·84	17·25	1·00

WL, weight loss; Wk9, week 9; Wk13, week13. Significant differences from baseline: * *P*<0·05, ** *P*<0·01, *** *P*<0·001.

† No differences between time points were seen.

### Adaptive thermogenesis

**Fig. 3:**
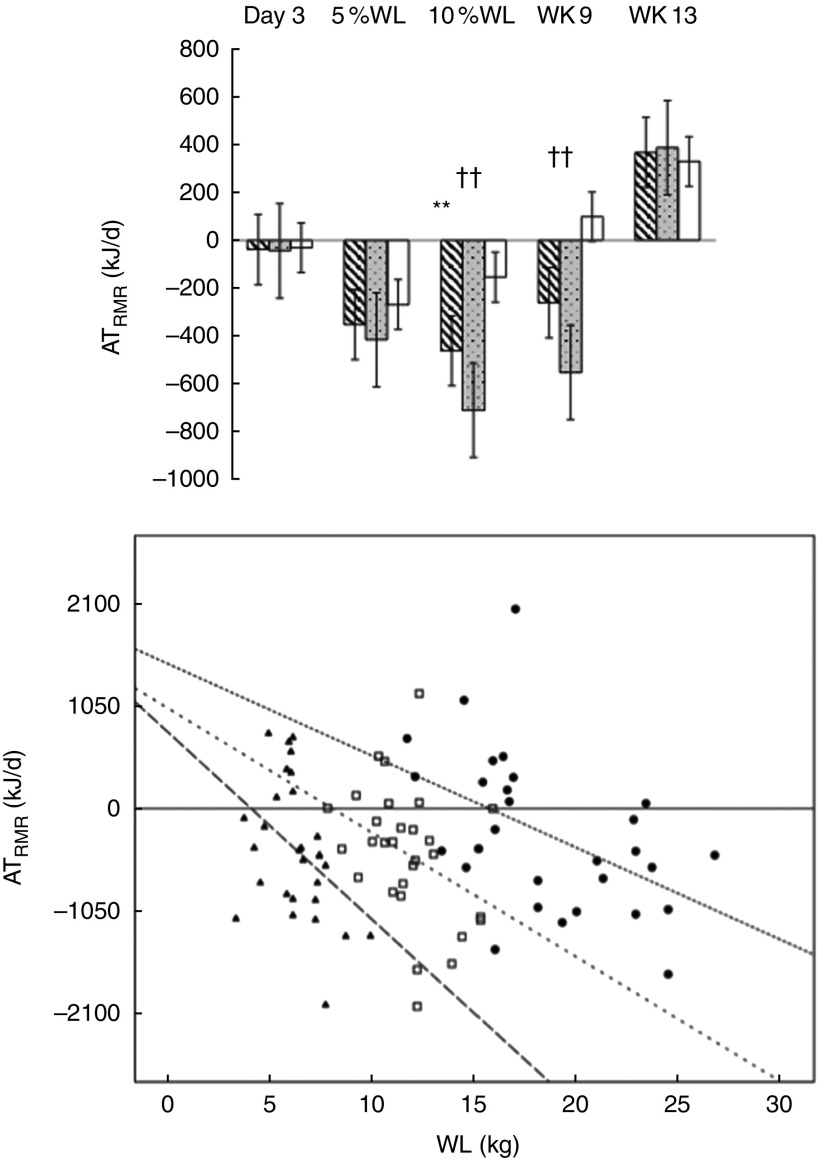
(a). Adaptive thermogenesis (AT) at the level of RMR with progressive weight loss (WL), in all participants (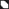), men (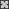) and women (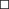). Values are means with their standard errors. Wk9, week 9; Wk13, week 13. RMR_measured_<RMR_predicted_: **P<0·01 for all, ††*P*<0·01 for males. (b). Correlation of AT_RMR_ against WL at 5 %, 10 % and week 9 in all participants was investigated by using Spearman’s *ρ* correlation coefficient; a larger AT_RMR_ was associated with a larger WL. The equation for the regression lines: 5 % WL; *Y*=−210×*X*+962, 10%WL; *Y*=−126×*X*+1025, and week 9; *Y*=−92×*X*+1483. 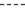, AT_RMR_ 5 % WL, *R* ^2^=0·241 (*P*<0·01); 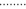, AT_RMR_ 10 % WL, *R* ^2^=0·153 (*P*<0·05); 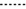, AT_RMR_ W9, *R* ^2^=0·285 (*P*<0·01).

AT_RMR_ was only significantly reduced after 10 % WL for all participants (−465 (SEM 691) kJ/d, *P*<0·01) and after 10 % WL and at Wk9 in men (−716 (SEM 670) and −553 (SEM 582) kJ/d, respectively, *P*<0·01 for both) (See Fig. 3(a)). AT_RMR_ was significantly higher in men compared with women at 5 and 10 % WL, and Wk9 (*P*<0·05, *P*<0·01 and *P*<0·05, respectively).

A negative correlation was found between magnitude of WL (kg) and AT_RMR_ at 5 % WL (*n* 30, *r* −0·491 and *P*<0·01), 10 % WL (*n* 29, *r* −0·391 and *P*<0·05) and Wk9 (*n* 29, *r* −0·224 and *P*<0·01), with a higher WL being associated with a larger AT_RMR_ (RMR_m_<RMR_p_) (Fig. 3(b)).

**Fig. 4:**
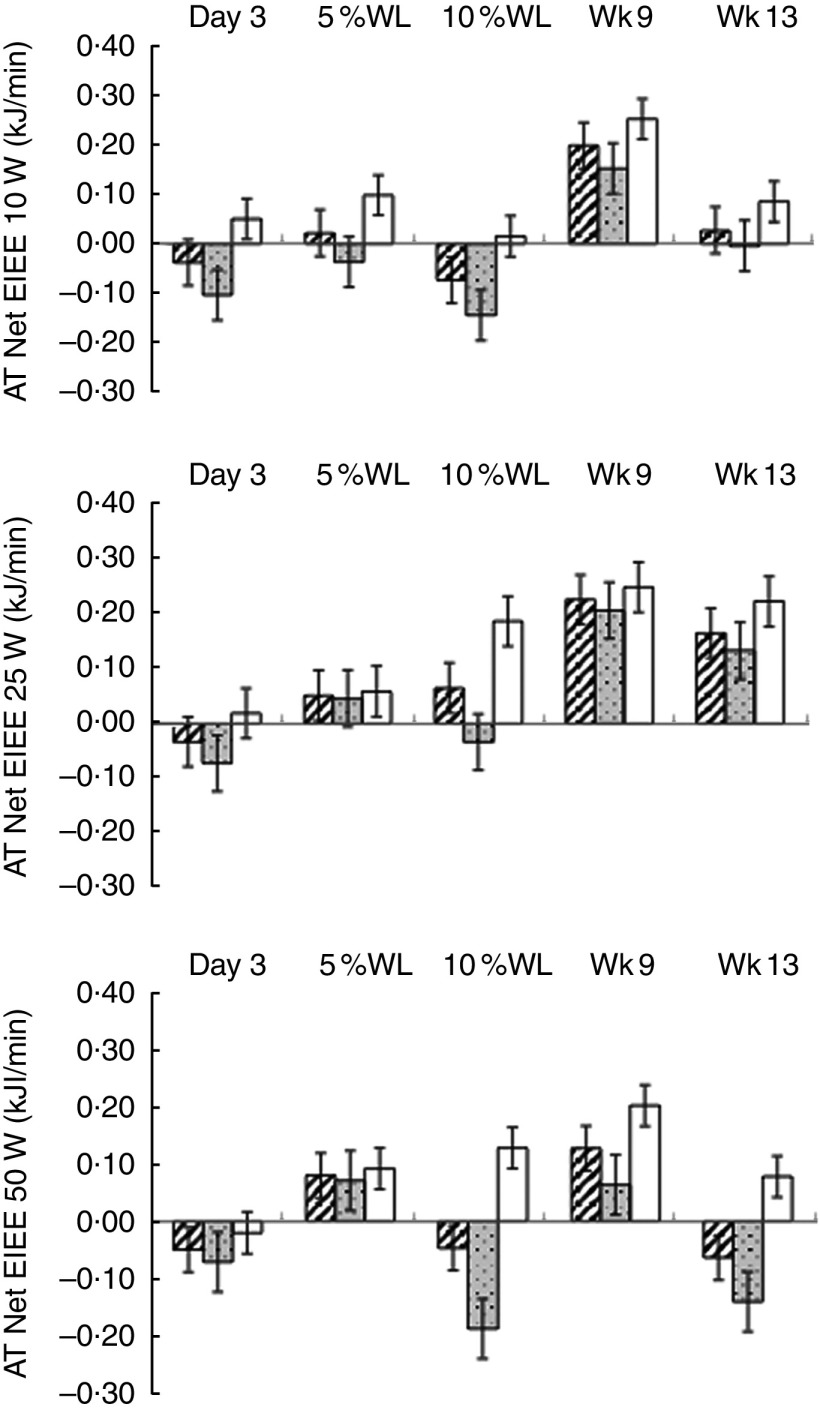
Adaptive thermogenesis (AT) at the level of net exercise-induced energy expenditure (EIEE) (10, 25 and 50 W) with progressive weight loss (WL) in all participants (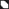), men (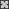) and women (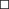). Values are means with their standard errors. Wk9, week 9; Wk13, week 13. No significant differences were found between net EIEE measured and predicted at any time point.

No evidence of AT_EIEE_ was found (Fig. 4) and AT_EIEE_ was not correlated with WL.

## Discussion

This study is the first to repeatedly measure RMR and EIEE, using a longitudinal design, to explore physiological adaptions to progressive WL. Despite significant WL by day 3 (2 (sem 1) kg WL), there was no significant drop in RMR, which is in line with previous research^(^ ^19^ ^)^. Our findings show a significant reduction in RMR (10 (sem 2) %) by the time 5 % WL (12 (sem 8) d) was reached, with no further reduction up to 16 % WL, which is in agreement with previous research^(^ ^33^ ^)^. Moreover, we found that the reduction in RMR was sustained even after a period of weight stabilisation, which again is in line with previous studies^(^ ^8^ ^,^ ^30^ ^)^.

A greater fall in RMR has been reported in studies with a shorter *v*. longer duration (≤ *v*. >6 weeks)^(^ ^34^ ^)^, suggesting that the reduction in RMR seen with WL is more pronounced during the 1st week of energy restriction, which supports our findings. When we adjusted RMR for FM and FFM, only a transient reduction was seen at 5 and 10 % WL in all participants and 10 and 16 % in men. This concurs with Ballor *et al*.^(^ ^35^ ^)^, who reported a significant reduction in RMR (both absolute and adjusted values) with a 10 % WL induced by diet alone. Contrary to our findings, Leibel *et al*.^(^ ^8^ ^)^ reported a reduction in RMR adjusted for FFM at 20 %, but not 10 % WL, in a mixed sample of men and women with obesity. Overall, our results show that a WL≥5 %, seen as the minimum required to achieve health benefits^(^ ^2^ ^)^, already leads to a significant reduction in RMR, but further WL up to 16 % does not induce a further significant decline in RMR.

The fact that we did not detect a significant change in EIEE until the WL was ≥10 % (12 (sem 2) kg) for all levels of power is in line with most of the available evidence^(^ ^10^ ^,^ ^20^ ^,^ ^35^ ^)^. However, Amati *et al*.^(^ ^22^ ^)^ did not find any change in gross EIEE (at an average power 38±2 W) with a 19 % WL induced by diet alone, followed by 2 weeks of weight stabilisation. Conflicting results may be explained by different protocols used to measure EIEE and the fact that this is sometimes expressed as gross and others as net EIEE.

AT_RMR_ was only present at 10 % WL in all participants and at 10 % WL and Wk9 (16 % WL) in men, which is similar to other studies^(^ ^30^ ^,^ ^36^ ^,^ ^37^ ^)^. Rosenbaum & Leibel^(^ ^37^ ^)^ reported AT_RMR_ after 10 %, but not 20 %, WL induced by diet alone. Camps *et al*.^(^ ^30^ ^)^ also found AT_RMR_ with 10 % WL achieved with a VLED, but opposite to us, that was sustained after 12 weeks of follow-up. On the other hand, other studies do not support the existence of AT_RMR_ after 5 or 10 % WL^(^ ^38^ ^,^ ^39^ ^)^. Differences in compliance, follow-up and sample size may account for some of these discrepancies. More studies are needed to confirm whether AT_RMR_ is indeed a transient phenomenon, and whether women are protected from it. In the present study, a larger AT_RMR_ was associated with a larger WL, up to 22 % WL, but not after a period of weight stabilisation. Camps *et al*.^(^ ^30^ ^)^ also found a correlation between AT_RMR_ and magnitude of WL, up to 25 % WL. In an in-patient, well-controlled study, Muller *et al.* ^(^ ^40^ ^)^ showed in eight normal-weight men a significant reduction in RMR, and the presence of AT_RMR,_ after only 3 d on a 50 % energy-restricted diet (WL, approximately 1·7 kg). They also showed a significant reduction in RMR and the presence of AT_RMR_ after 1 week (WL, approximately 2·2 kg) in 32 non-obese men, with no further significant changes with progressive WL up to 3 weeks (WL, approximately 4 kg (5 % WL)). Inconsistencies in outcomes between this and the present study may be owing to differences in sex distribution (males *v*. mixed sex), participant’s characteristics (non-obese *v*. obese), dietary intervention and magnitude of WL. Even though the accuracy of our RMRp was not perfect, it is in line with that seen with WHO equation^(^ ^41^ ^)^ and we were unable to find any established equation that would result in a better accuracy.

No AT_EIEE_ was found at any time point or level of power in the present study. Other studies have reported AT_EIEE_ to be present after a WL between 10 and 20 %, followed by 2–3 weeks of weight stabilisation^(^ ^8^ ^,^ ^9^ ^)^. Differences in outcomes among studies can probably be explained by diverse sample sizes, participants’ characteristics, magnitude of WL, WL intervention and protocols used to measure and predict EIEE (stationary bike *v*. treadmill, different resistances, speeds and inclinations). Moreover, non-resting EE is not the same as EIEE, and thus comparisons between studies need to be done carefully. When adjusting RMR for body composition, and assessing the presence of AT_RMR_, it was assumed that the composition of FFM was constant during WL. However, FFM hydration probably changed, given that ketogenic diets lead to a large loss of total body water, owing to glycogen depletion, during the 1st days of the diet^(^ ^42^ ^,^ ^43^ ^)^. This might have biased body composition results and affected our outcomes, particularly those taken at day 3. These results need to be interpreted with caution, given that the accuracy of EIEEp was not optimal. However, we are not aware of any established equation that could be used to improve the accuracy of EIEEp.

This study revealed some important sex differences. In women, there was no significant change from baseline in neither absolute nor adjusted RMR, after a 16 % sustained WL. This is in line with Doucet *et al*.^(^ ^44^ ^)^, who found a sustained reduction in RMR after an average 10 % WL only in men. On the other hand, Schwartz & Doucet^(^ ^34^ ^)^, in a systematic review on the effects of diet-induced WL on RMR, reported a similar decrease in RMR for both sexes. A reduction in net EIEE in women was only seen for 10 W at Wk13 (16 (sem 2) %), which is in line with a previous study^(^ ^14^ ^)^. Some of the sex differences seen in this study may be attributed to differences in energy and protein deficit, which lead to a larger overall WL in men and might have also contributed to the fact that FFM did not change in women, whereas in men there was a significant reduction at 16 % WL. This is supported by literature, which suggests that FFM reduction during WL is proportionally greater in men^(^ ^23^ ^)^. AT_RMR_ was not seen in women at any time point. This is in line with Doucet *et al*.^(^ ^13^ ^)^, who reported AT_RMR_ after 8 weeks on a diet in men only^(^ ^13^ ^)^. On the other hand, Camps *et al*.^(^ ^30^ ^)^ reported AT_RMR_ in both sexes after a 9·6 (sem 4·1) kg WL induced with a VLED. Leptin has been suggested as a potential mediator to explain the differences in AT between sexes^(^ ^45^ ^)^. Owing to their relatively higher percentage of FM compared with men, women have a higher leptin plasma concentration, and the reduction seen with WL may translate in leptin plasma concentration falling below a threshold level in men, but not in women^(^ ^46^ ^)^. Given that AT_RMR_ has been shown to be positively correlated with the reduction in leptin seen with WL^(^ ^47^ ^)^, it could potentially explain why AT_RMR_ was only seen in men in this study.

This study has several strengths. First, its longitudinal design is unique, with multiple measurements undertaken during progressive WL. This allowed us to evaluate the effect of minimal, but significant WL (day 3), WL known to induce health benefits (5–10 %)^(^ ^2^ ^)^ and a larger WL (16 %), before and after weight stabilisation, on the different outcome variables. Second, compliance was objectively monitored and was excellent. Third, we adjusted for multiple comparisons and multiple outcome variables. However, this study has also limitations. The fact that body composition was measured by ADP, and as such did not take into account the level of FFM hydration, may have affected the absolute values, particularly regarding adjusted RMR and AT at rest. Moreover, the best regression model to predict EIEE had a relatively modest *R* ^2^, with <47 % of the variation in EIEE being explained by the model, which could have an impact on our estimation of AT_EIEE._ It needs also to be acknowledged that measured baseline RMR values in this study were on average 20 % lower than predicted by the Mifflin equation^(^ ^48^ ^)^, with 80 % of the participants presenting RMR values below predicted (difference between measured and predicted >10 %). This has been previously described. Weijs^(^ ^49^ ^)^ showed in an adult Dutch population with overweight and obesity (average BMI 30 kg/m^2^) that 50 % of the individuals had a measured RMR (measured with the same equipment as in the present study) lower than that predicted by the Mifflins equation. The reasons for the lower percentage of individuals with accurate values in the present study compared with Weijs remains speculative, but the fact that our population was substantially more obese (BMI 36·7 (sem 4·5) *v*. 30·8 (sem 3·6) kg/m^2^), had a body composition with a high % of non-metabolically active body fat and also with different genetic background (Norwegian) might all have had an impact. Moreover, our sample comprises individuals with obesity who had sought treatment, and it is possible that at least some of the participants presented with AT as a result of previous weigh loss–regain cycles^(^ ^17^ ^)^. As the main aim of this study was to look at changes over time, even if RMR values are underestimated compared with a standard predictive equation, the longitudinal nature of the study methods and statistical analysis takes into consideration baseline values, and thus the overall findings of the study are still valid. Finally, the study may be underpowered to examine sex differences.

This study has several practical implications. Patients need to be assured that a WL >5 %, and up to 16 %, will not necessarily translate into further significant reductions in their basal energy needs. This knowledge is important for practitioners when reformulating dietary prescriptions for progressive WL and WL maintenance. When aiming for progressive weight reduction after ≥10 % WL, a larger dietary energy restriction and/or an increase in PA levels are essential to counteract the decrease in EIEE seen at this time point. A slowdown in WL rate after ≥10 % WL can, at least partially, be explained by the transient AT_RMR_ seen at this time point. Practitioners need to be aware of these physiological adaptations and not assume that non-compliance with the diet is the sole explanation. Knowing when changes in EE, at rest and during exercise, occur with progressive WL is important to understand resistance with progressive WL and relapse after treatment, and should be discussed alongside patients’ expectations of their WL journey.

In conclusion, a fall in RMR should be anticipated at ≥5 % WL, a reduction in EIEE at ≥10 % WL and transient ATRMR at 10 % WL. These metabolic compensatory responses can make further WL difficult and increase the risk of relapse. Sex seems to modulate some of these responses, but larger long-term longitudinal studies are needed.
